# Advances and perspectives on perylenequinone biosynthesis

**DOI:** 10.3389/fmicb.2022.1070110

**Published:** 2022-12-20

**Authors:** Huaxiang Deng, Xinxin Liang, Jinbin Liu, Xiaohui Zheng, Tai-Ping Fan, Yujie Cai

**Affiliations:** ^1^Center for Synthetic Biochemistry, Shenzhen Institute of Synthetic Biology, Shenzhen Institute of Advanced Technology, Chinese Academy of Sciences, Shenzhen, China; ^2^The Key Laboratory of Industrial Biotechnology, Ministry of Education, School of Biotechnology, Jiangnan University, Wuxi, Jiangsu, China; ^3^School of Marine and Bioengineering, Yancheng Institute of Technology, Yancheng, Jiangsu, China; ^4^College of Life Sciences, Northwest University, Xi’an, Shanxi, China; ^5^Department of Pharmacology, University of Cambridge, Cambridge, United Kingdom

**Keywords:** perylenequinones, pathway deciphering, metabolite orchestration, metabolite platform, automatic engineering, high throughput tools

## Abstract

Under illumination, the fungal secondary metabolites, perylenequinones (PQs) react with molecular oxygen to generate reactive oxygen species (ROS), which, in excess can damage cellular macromolecules and trigger apoptosis. Based on this property, PQs have been widely used as photosensitizers and applied in pharmaceuticals, which has stimulated research into the discovery of new PQs and the elucidation of their biosynthetic pathways. The PQs-associated literature covering from April 1967 to September 2022 is reviewed in three sections: (1) the sources, structural diversity, and biological activities of microbial PQs; (2) elucidation of PQ biosynthetic pathways, associated genes, and mechanisms of regulation; and (3) advances in pathway engineering and future potential strategies to modify cellular metabolism and improve PQ production.

## Introduction

Perylenequinones (PQs) comprise a basic 4, 9-dihydroxy-3, 10-PQ structure (Supplementary Figure S1; Daub et al., 2013). They can be substituted in position 2, by two methoxy groups, in positions 1 and 12 by a six-or seven-membered ring, or by two 2-hydroxy-or 2-acyloxy-propyl side chains, and in positions 6 and 7 by a methylenedioxy group, or by two methoxy groups (Daub et al., 2013). These PQs have been divided into four classes based on the substitutional groups (Geris et al., 2022). The PQs progresses of class A, C and D have been reviewed previously (Falk, 1999; Barnes et al., 2001; Miskovsky, 2002; Dumas et al., 2004; Saw et al., 2008; Geris et al., 2022). Therefore, this review is focused on the class B perylenequinones.

In the presence of light, PQs are photoactivated and catalyze the formation of reactive oxygen species (ROS) from singlet oxygen. Excessive ROS can disrupt the balance of cellular redox status and cause oxidative stress, thereby causing oxidative damage to cellular macromolecules, resulting in the inhibition of metabolism and cellular apoptosis in pathogenic microorganisms and cancer cells (Qi et al., 2019; Wang et al., 2019). Consequently, PQs have been widely applied as photosensitizers in the medical, food, cosmetic, agricultural, and material fields (Table 1; Gao et al., 2012; Zheng et al., 2018).

**Table 1:** Functions and original strains of diverse perylenequinones.

Chemicals	Functions*	Strains	References
Phaeosphaerins A-F	Potentially kill human tumor cells	*Phaeosphaeria* sp.	Li et al. (2012)
Calphostin and the derivatives	Induce cell apoptosis; exhibit the activity against Protein kinase C and HIV; display antimicrobial and antitumor activity	*Cladosporium* species; *Phaeosphaeria* sp.	Kaul and Maltese (2009), Li et al. (2012), and So et al. (2018)
Cercosporin and the derivatives	Exhibit antileishmanial activity and cytotoxicity for diverse cells, including glioblastoma multiforme, breast adenocarcinoma, and pig kidney epithelial cells; Biosynthesize medical chemicals	*Cercospora* species; *Scolecotrichum graminis* Fucke; *Septoria pistaciarum*; *Colletotrichum fioriniae*	Tabuchi et al. (1991), Tabuchi et al. (1994), Kumarihamy et al. (2012), de Jonge et al. (2018), Grigalavicius et al. (2019), and Mastrangelopoulou et al. (2019)
Elsinochrome and the derivatives	Exhibits the cytotoxicity to cell L929 and KB3.1; inhibit growth of diverse microorganisms, such as *Staphylococcus aureus*	*Parastagonospora nodorum*; *Stagonospora convolvuli* LA39; *Shiraia* sp.	Boss et al. (2007), Cai et al. (2011), and Surup et al. (2018)
Hypocrellins and the derivatives	Cure skin diseases, rheumatoid arthritis, gastric diseases; induce apoptosis of diverse cancer cells; display the inhibition activity of SARS-CoV-2	*Hypocrella bambuase*; *Shiraia bambusicola*; *Penicillium chrysogenum*; *Phaeosphaeria* sp.	Diwu and Lown (1990), Li et al. (2012, 2021), and Meng et al. (2011)

The high demand for PQs has stimulated research into improving their production and purity, such as sequencing of the potential PQ biosynthesis gene clusters (BGCs), which have served as sequence libraries for improving and diversifying PQ biosynthesis of the full range of PQs, and has improved PQ yields in many instances (Blin et al., 2017). However, no systematic review of PQs is available. To fill this gap, this comprehensive review covers progress in PQ research in three sections: (1) the sources, structural diversity, and biological activity of microbial PQs, (2) elucidation of PQ biosynthetic pathways, associated genes and mechanisms of regulation, and (3) advances in pathway engineering and future potential to modify cellular metabolism and improve PQ production.

## Microbial perylenequinones, structural diversity and bioactivity

The five cycle structures (Supplementary Figure S1) contribute to the specific PQs bioactivities against diverse fungi, bacteria, and cancer cells (Kobayashi et al., 1989b). The subtle structure might endow particular bioactivities of PQs (Diwu and Lown, 1990; Surup et al., 2018). The above phenomenon inspires scientists to exploit the corresponding microorganisms and functions of diverse PQs.

### Phaeosphaerins

As cytotoxic PQs, phaeosphaerins can be photoactivated to generate ROS, which accumulates in the lysosomes of human tumor cells and induces apoptosis of these cells. Phaeosphaerins A-F **(1–5)** have been isolated from *Phaeosphaeria* sp. (Supplementary Figure S2; Li et al., 2012).

### Calphostin and derivatives

Calphostin A, B, C, D, and I (**6–10**; Supplementary Figure S3) have inhibitory activity against protein kinase C (PKC), and Calphostin C is the strongest inhibitor (Kobayashi et al., 1989b). Calphostin C induces cell apoptosis, both *via* the dual leucine zipper-bearing kinase and c-Jun N-terminal kinase (JNK) signaling pathways (Robitaille et al., 2008) and by inducing endoplasmic reticulum stress (Kaul and Maltese, 2009). Calphostin C also inhibits the expression of *Chlamydophila pneumoniae*-associated intracellular adhesion molecule-1 and blocks NF-kappaB translocation (Vielma et al., 2003a,b). These potentially useful biological activities have stimulated extensive research aimed at identifying alternative sources of Calphostins and improving their biosynthetic efficiency. For instance, Calphostins were first isolated from *Cladosporium cladosporioides* culture medium, during screening for protein kinase C (PKC) inhibitors (Iida et al., 1989; Kobayashi et al., 1989a), and other *Cladosporium* species can also produce calphostin derivatives. In addition, *C. cucumwinum* from etiolated cucumber seedlings produces Cladochrome A (**12**; Overeem et al., 1967) and *C. cladosporioides* can biosynthesize diverse PQs after epigenetic modifier treatment, including Cladochromes A **(12)**, B **(13)**, D **(14)**, E **(15)**, *F* **(16)**, G **(17)**, and calphostin B (Williams et al., 2008). *Phaeosphaeria* sp. also produces (+)-calphostin D (Li et al., 2012). The homologous PQs to calphostin, phleichrome **(18)** and isophleichrome **(19)** have been isolated and characterized from the plant pathogens *C. phlei* and *C. herbarum* (Yoshihara et al., 1975; Robeson and Jalal, 1992). Isophleichrome is also produced by *C. cucumwinum* in low yield (Arnone et al., 1989). These phleichromes are stronger inhibitors of HIV-1 and PKC than calphostin, and have antimicrobial, and antitumor activities based on their photodynamic properties (So et al., 2018).

### Cercosporin and derivatives

Cercosporin **(20)** is another well-known photoactivated PQ toxin from diverse *Cercospora* species (Supplementary Figure S3). These phytopathogens cause leaf spot and blight diseases on diverse crop species (Daub, 1981; Daub and Ehrenshaft, 2000). Cercosporin also has antileishmanial activity and cytotoxicity for glioblastoma, breast adenocarcinoma, and human tumor cell lines (Kumarihamy et al., 2012; Grigalavicius et al., 2019; Mastrangelopoulou et al., 2019). Photoactivation of cercosporin has applications in the biosynthesis of medicinal compounds. For instance, the cercosporin-photocatalyzed sp^3^(C-H) activation reaction can be used to synthesize pyrrolo[3,4-c]quinolones, which are the backbone structures of various bioactive compounds, such as “A Disintegrin and Metalloproteinase with Thrombospondin Motif” (ADAMTS) inhibitors (Li et al., 2019). The cercosporin-photocatalyzed oxidation reaction can be also employed to synthesize different kynurenine derivatives and the relevant peptides, which present the neuroprotective bioactivity (Yuan et al., 2022). The great pharmacological value of cercosporin and its derivatives has stimulated their isolation from diverse microorganisms. *Cercospora kikuchii* was the first reported cercosporin producer (Kuyama and Tamura, 1957) and at least 24 other *Cercospora* species can produce cercosporin (Assante et al., 1977). Various derivatives of cercosporin with modified functional groups have been also isolated, such as (+)-isocercosporin **(21)** from *Cercospora kikuchii*, and (+)-Isocercosporin and acetylisocercosporin **(22)** from the plant pathogen *Scolecotrichum graminis* Fuckel (Tabuchi et al., 1994). *Septoria pistaciarum* produces (+)-Cercosporin, (+)-14-O-Acetylcercosporin and (+)-di-O-Acetylcercosporin (Kumarihamy et al., 2012). In a study characterizing the cercosporin toxin biosynthesis (CTB) gene cluster in the phytopathogenic genus *Cercospora*, de Jonge et al. also found that treatment of *Colletotrichum fioriniae* with trichostatin A stimulates the production of a cercosporin-associated PQ (de Jonge et al., 2018), thus indicating that *Cercospora* has the potential to produce cercosporin-associated PQs.

### Elsinochrome and derivatives

Elsinochrome A is another well-known photoactivated PQs toxin from the phytopathogenic *Elsinoe* species (Supplementary Figure S3; Weiss et al., 1957). Similarly to Cercosporin, Elsinochrome A is cytotoxic to the mouse fibroblast cell line L929 and human HeLa cell line KB3.1 (Surup et al., 2018). Elsinochrome A also has antimicrobial activity against *Bacillus subtilis*, *Mucor hiemalis*, and *Staphylococcus aureus* (Surup et al., 2018). Further research on Elsinochrome-producing microorganisms resulted in the isolation of Elsinochromes A, B, C, and D **(23–26)** from other *Elsinoe* species (Batterham and Weiss, 1963; Lousberg et al., 1969, 1970). Other microorganisms can also produce Elsinochrome, for example, Elsinochrome C is produced by *Parastagonospora nodorum* (Chooi et al., 2017). The mycobiont fungal strain of the lichen *Graphis elongata* biosynthesizes Elsinochrome A (Fazio et al., 2018). *Hypomyces* sp., a parasitic mushroom fungus, produces hypomycin A **(27)** and B **(28)**, with structures closely related to the elsinochromes (Liu et al., 2001; Zhang et al., 2001). It is noted that *Stagonospora convolvuli* LA39 can produce both elsinochrome A and cercosporin (Boss et al., 2007). *Shiraia* sp. SUPER-H168 produces a PQ complex of elsinochrome and hypocrellin (Cai et al., 2011), thus indicating that diverse microorganisms contain the PQ biosynthetic gene clusters (de Jonge et al., 2018).

### Hypocrellin and derivatives

As an effective defense compound against disease, hypocrellin has been used for centuries in traditional Chinese medicine, to effectively treat skin diseases, rheumatoid arthritis, and gastric diseases (Diwu and Lown, 1990). Hypocrellin A **(29)** and B **(30)** present excellent antimicrobial and antileishmanial activities (Ma et al., 2004) and can induce apoptosis of diverse cancer cells, such as hepatocellular carcinoma (Wang et al., 2019), human lung adenocarcinoma A549 cells (Qi et al., 2019), and squamous carcinoma A431 cells (Niu et al., 2020). Hypocrellin can also protect human ACE2 cells against infection by SARS-CoV-2 by blocking the receptor-binding domain of the SARS-CoV-2S protein, thereby inhibiting the entry of the virus into human cells (Li et al., 2021). Given its potential as a SARS-CoV-2 entry inhibitor, hypocrellin and its derivatives have been isolated from diverse microorganisms (Supplementary Figure S3) to improve the supply of hypocrellin, which was first isolated from the stroma of *Hypocrella bambuase,* growing on bamboo shoots (Chen et al., 1981). *Shiraia bambusicola,* another bamboo parasitic fungus, is also able to biosynthesize hypocrellins (Liu, 1985), as are *Penicillium chrysogenum* and *Phaeosphaeria* sp. (Meng et al., 2011; Li et al., 2012) and the engineered *Aspergillus* species (Hu et al., 2019).

## Elucidating the perylenequinone biosynthetic pathway

The widespread applications of PQs have prompted considerable research efforts to elucidate and exploit their biosynthetic pathways for industrial production (de Jonge et al., 2018; Hu et al., 2019). Initially, bioinformatic methods, such as genome walking and genome evolutionary analysis were used to screen for potential PQ gene clusters (Chooi et al., 2017; de Jonge et al., 2018; Deng et al., 2020b). Then these candidate PQs biosynthetic genes were verified by gene disruption and chemical structure comparison between wild-type strains and mutants (Deng et al., 2017a; Hu et al., 2019). *In-vitro* enzymatic assays were used to confirm the gene functions by heterologous expression of the relevant genes in suitable hosts, enzymatic conversion of substrates and, determination of structural differences among substrates, intermediates, and products (Newman and Townsend, 2016).

### PQ pathway discovery by gene cluster conservation analysis

**Figure 1:**
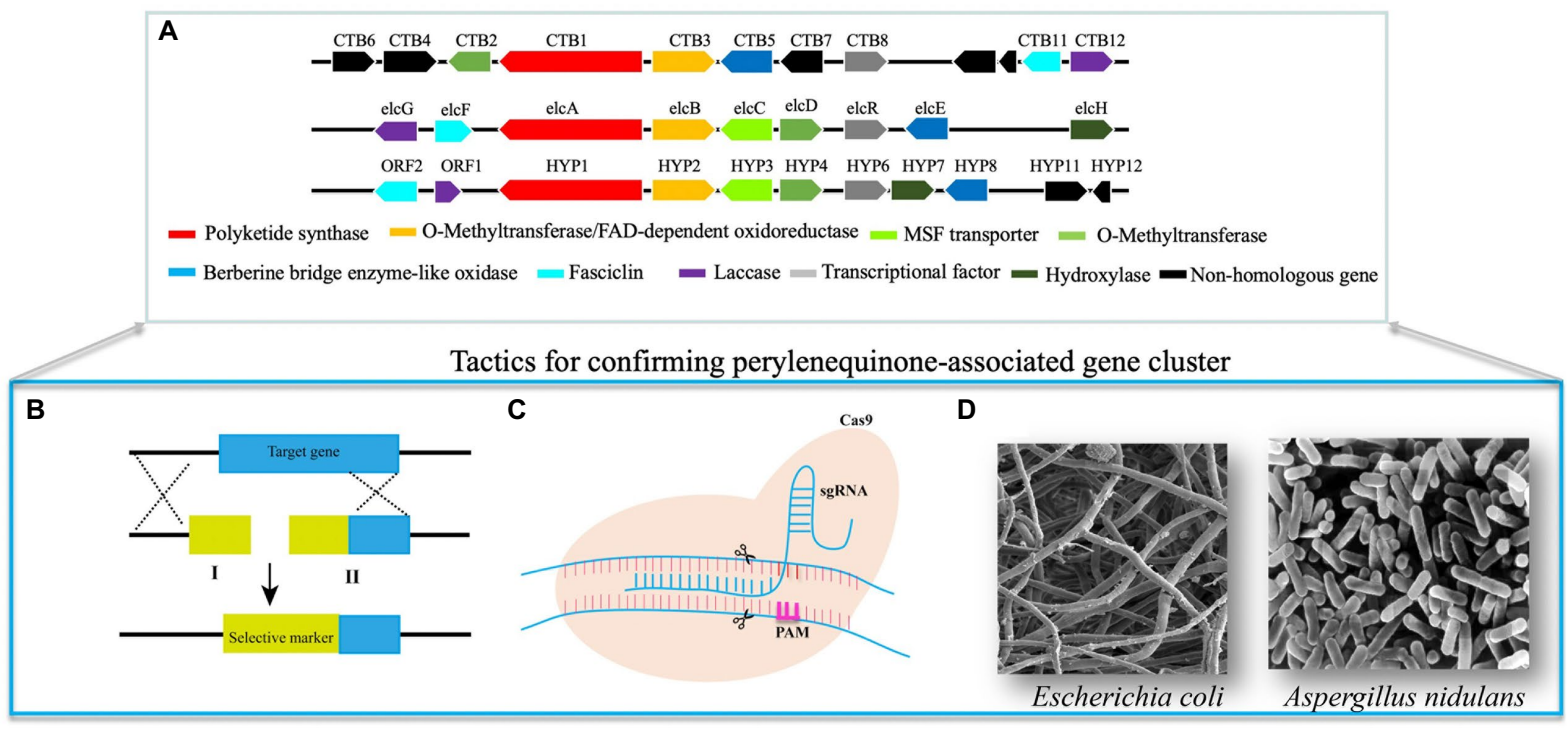
Methods for confirming perylenequinone-associated genes and pathways. **(A)** Homologous PQs-associated biosynthetic gene clusters. **(B)** For this split-marker approach, we need to synthesize two nucleotides fragment I and II. Fragments I comprise a partial selective marker and a sequence homologous to 5′ target locus; fragments II contain a partial selective marker and a sequence homologous to 3′ target locus. Thus, 5′ and 3′ homologous arms facilitate relevant homologous recombination of target genes and mutants can be screened on antibiotic regeneration plates due to the overlapping sequences of the selective marker of two fragments. **(C)** The CRISPR system contains two crucial elements, including Cas9 nuclease and sgRNA. The complex of these two elements precisely triggers double-strand breaks, induces cellular repair pathways, and results in relevant gene modification. **(D)** Verification of crucial PQs-associated genes by expressing them in the tractable microorganisms and then following chemical comparisons.

**Figure 2:**
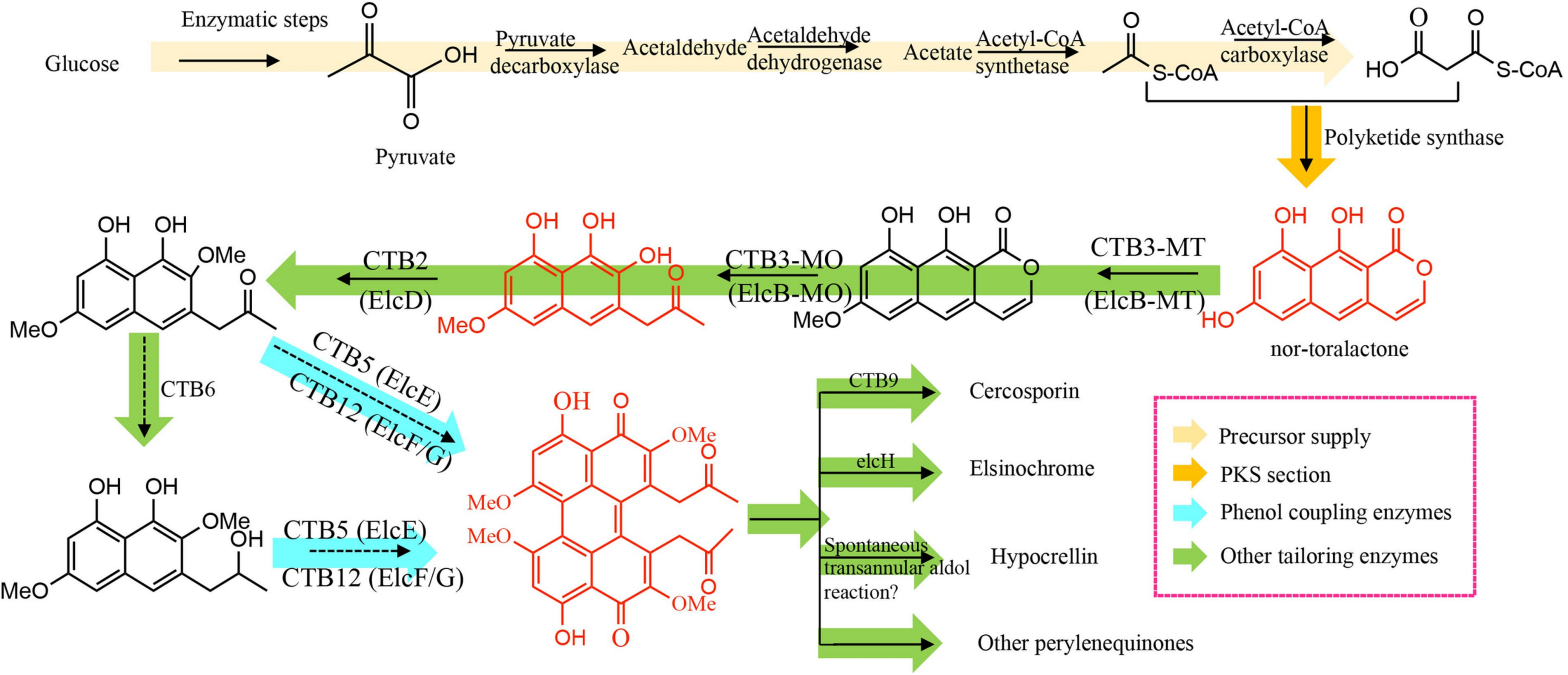
Schematic representation of perylenequinone flux using glucose as the original substrate.

Genes for PQ biosynthesis are arranged as biosynthetic gene clusters (BGCs; Blin et al., 2017), comprising three modules: i.e., the polyketide synthase (PKS), which synthesizes the core PQ structure; phenol coupling enzymes, which dimerize the core structure; and tailoring enzymes, which modify the functional groups and generate the diversity of PQ structures (Müller et al., 2019; Figures 1A, 2). This collinear BGC structure has facilitated the discovery of new PQs by homology analysis. For example, the elsinochrome BGC from *Elsinoë fawcettii* and the phleichrome BGC from *C. phlei* have been identified through homology analysis of the cercosporin and hypocrellin BGCs, respectively (Ebert et al., 2019; Hu et al., 2019).

The PKS enzymes in different PQ biosynthetic pathways are characterized by high sequence homology and structural conservation. Therefore, using PKS as a screening template is an effective approach to discover potential new PQ BGCs. For example, our group has elucidated and confirmed the hypocrellin pathway in *S. bambusicola*, based on homology analysis of the cercosporin and elsinochrome PKSs (Deng et al., 2017a). A genome evolutionary method has been developed to mine PQ BGCs through PKS homology analysis, based on the collinearity of BGCs (de Jonge et al., 2018), which has been used to discover that the cercosporin BGC has undergone multiple duplications and horizontal transfers across a wide range of plant pathogenic fungi. Three cercosporin BGCs were found in the *Cercospora beticola* genome, two of which shared the major cercosporin-associated genes. A “phylogenetic roadmap” of the putative evolutionary history of polyketide synthase (PKS) was also created (de Jonge et al., 2018), which showed that four duplications and three transfers of the cercosporin PKS had occurred from diverse *Dothideomycetes* and *Glomerellales* species. The identity of the proposed cercosporin PKS remains to be confirmed by gene knockout and chemical characterization of its substrate selectivity/products.

### Confirmation of perylenequinone-associated genes through gene disruption

Knockout of a PQ biosynthesis gene may decrease or eliminate the related enzymatic function (Liu et al., 2022). Phenotypical and metabolic differences between wild-type strains and mutants can aid in the elucidation of gene functions, for example, the pentacyclic core structure of PQs confers a variety of colors (Hu et al., 2019; Liu et al., 2022). Wild-type strains are mostly red, whereas mutants exhibit various other colors, owing to the accumulation of biosynthetic intermediates (Newman and Townsend, 2016). This aspect has greatly facilitated the discovery of the specific functions of the essential PQ biosynthetic genes.

#### Gene knockout by conventional homologous recombination

The split-marker approach is a common genetic manipulation to verify the functions of PQ-associated genes (Figure 1B; You et al., 2009). Based on the lengths of homologous donors fused with selective markers (Figure 1B), the gene targeting efficiency of split-marker technology can be up to 50%. In addition, this approach can also decrease ectopic integration and enhance the corresponding genetic modification. Split marker methods have generated diverse mutants of genes in the PQ pathway (Chen et al., 2007; You et al., 2009; Newman and Townsend, 2016), resulting in metabolite differences between wild-type and mutant strains. For example, the PKS and its transcription factor (TF) in the elsinochrome pathway have been disrupted by the split marker method (Chung and Liao, 2008; Liao and Chung, 2008). The relevant mutants lose the elsinochrome production ability, and the adjacent genes in these mutants show absent or diminished transcription. Deletion of a MAP kinase homolog gene (*CZK3*) also suppresses cercosporin production in the relevant mutant (Shim and Dunkle, 2003). The red pigment around the mycelia of wild-type *Cercospora zeae-maydis* was abolished, resulting in the colorless ∆CZK3 mutant (Shim and Dunkle, 2003). The other eight genes in the cercosporin pathway have also been individually deleted (Chen et al., 2007). Phenotypic analysis has indicated that mycelia of the cercosporin toxin biosynthesis (∆CTB1) mutant lacks any color pigments, whereas the ∆CTB2, ∆CTB3c, and ∆CTB7 mutants are a yellow-brown color, and ∆CTB5 and ∆CTB6 mutants are a dark orange-red color (Newman and Townsend, 2016). Nor-toralactone, toralactone and naphthoquinone have been produced by the ∆CTB3c mutant, suggesting that CTB3c’s functions include the opening cycle, decarboxylation, and hydroxylation (Newman and Townsend, 2016). The ∆CTB6 mutant produces cercoquinone A, which lacks the O-methyl group of the wild-type quinone compound, thus suggesting that CTB6 is involved in the reduction reaction (Newman and Townsend, 2016). In contrast, the ∆CTB1, ∆CTB2, and ∆CTB7 mutants produce no major metabolites and the ∆CTB5 mutant produces cercoquinone B. No cercosporin is detected in the ∆CTB9 mutant and the following *in-vitro* assay further reveals that CTB9 catalyzes the methylenedioxy bridges of cercosporin (Liu et al., 2022).

#### Gene knockout by CRISPR

Compared with conventional gene knockout tools, the clustered regularly interspaced short palindromic repeats (CRISPR) system is simple, inexpensive and high-throughput (Deng et al., 2020a), and has been widely used for genetic modification of diverse organisms (Deng et al., 2017b). The Cas9 nuclease and sgRNA are two critical elements in the CRISPR system. The complex of Cas9 and sgRNA triggers specific double-strand breaks, induces cellular repair pathways, and modifies the target genes (Sander and Joung, 2014; Figure 1C). Our group has recently constructed a CRISPR system in *S. bambusicola* and generated several mutants (Deng et al., 2017a,c, 2018), including mutants of TF, polyketide synthase, monooxygenase, and a major facilitator superfamily transporter, all of which decrease hypocrellin production. Compared with the red wild-type *S. bambusicola*, transcription factor and polyketide synthase mutants are colorless, whereas mutants of monooxygenase and major facilitator superfamily transporter are brown. None of these mutants could produce hypocrellin. Relative expression experiments have not detected transcription of adjacent genes (Deng et al., 2017a, 2020b), or the presence of their corresponding intermediates (Deng et al., 2018). These findings remain to be confirmed by heterologous expression of these enzymes and substrate selectivity/product characterization.

### Confirmation of PQ-linked gene functions by heterologous expression in host microorganisms

Sometimes, gene disruption mutants can generate no, or ambiguous metabolites, thus hampering the confirmation of their functions. To avoid this situation, the corresponding enzymes can be heterologously expressed in optimized host systems, such as *Escherichia coli* and *Aspergillus* species, and their products can be characterized (Figure 1D).

*Escherichia coli* has several advantages as a host for gene expression, such as rapid growth rate, high protein expression levels, and well-constructed genetic tools, which has made it the expression system of choice for PQ genetic studies. However, polyketide synthases, the first enzyme in PQ biosynthesis, comprise 2,000–3,000 amino acids, with starter unit acyltransferase (SAT), β-ketoacyl synthase (KS), malonyl acyltransferase (MAT), product template (PT), dual-tandem acyl-carrier protein (ACP_2_), and thioesterase (TE) domains (Herbst et al., 2018). These large, multi-domain enzymes cannot be expressed efficiently in *E. coli*, so the Udwary-Merski algorithm (UMA) was designed to predict the locations of individual domains and the linker regions between them (Udwary et al., 2002). This allows the domain structure of the gene to be deconstructed, and individual domains can be expressed in *E. coli*, then purified (Crawford et al., 2008); followed by combining the various domains with substrates and analyzing the PQ products. When the PKS gene was subjected to this analysis, the tri-domain SAT-KS-MAT was expressed in soluble form, but not KS-MAT (Crawford et al., 2008; Herbst et al., 2018), thus indicating that the tri-domain SAT-KS-MAT can assemble as a substrate loading/condensation complex, yielding a heptaketide. Through the same approach, the PT domain is involved in the C4-C9 and C2-C11 cyclizations by this heptaketide (Newman et al., 2014), and the TE domain has been identified to contribute to pyrone formation (O13-C1 cyclization) and nor-toralactone release (Newman et al., 2012).

Unfortunately, codon usage bias and species differences often result in insoluble, or unstable proteins when the bacterial *E. coli* expression system is used for fungal enzyme expression, thus restricting the utility of the deconstruction approach. However, these problems can often be avoided by using fungal expression systems, such as *Aspergillus* (Pahirulzaman et al., 2012; Chiang et al., 2013), which has facilitated the complete elucidation of the PQ biosynthetic pathway. For example, the *Cppks1* gene from *C. phlei* has been expressed in *Cryphonectria parasitica*, thereby facilitating the elucidation of the biosynthetic pathway of the red phleichrome pigment (So et al., 2015). Engineered filamentous fungi such as *Penicillium crustosum* and *Aspergillus* species were successfully transformed and enabled to produce nor-toralactone (Chooi et al., 2017; Hu et al., 2019; Kindinger et al., 2019).

The availability of the PQ precursor nor-toralactone has enabled the verification of other PQ pathway genes (Figure 2); for example, the O-methyltransferase domain of the di-domain enzyme CTB3 O-methylates nor-toralactone (Newman and Townsend, 2016). The monooxygenase domain of CTB3 catalyzes the opening cycle, decarboxylation, and hydroxylation (Newman and Townsend, 2016); similarly, the di-domain protein in the elsinochrome pathway has similar functions (Hu et al., 2019). Engineered *A. nidulans* with elcA and elcB-MT transformed nor-toralactone to toralactone, suggesting the O-methylation function of elcB-MT. Co-expression of elcA and elcB can also convert toralactone to three different products, including one with a molecular mass consistent with cercoquinone A and D (Newman and Townsend, 2016). This finding indicates that elcB is involved in O-methylation, the opening cycle, decarboxylation, and hydroxylation.

It appears that an FAD-linked oxidase is involved in dimerizing the phenolic precursor to the corresponding PQ. However, a laccase-like multicopper oxidase (ElcG) can also catalyze dimerization by combining with a berberine bridge enzyme-like oxidase (ElcE; Hu et al., 2019). The FAD-dependent CTB5 from *Cercospora nicotianae* and Cz_CTB12 from *Cercospora zeae-maydis* appear to synergistically catalyze the dimerization reaction. In addition, a multicopper oxidase has been identified, which can biosynthesize the atropisomer (*P*)-viriditoxin by dimerizing two regionally-selective semiviriditoxin molecules (Hu et al., 2019). A fungal laccase was determined to regionally dimerize the phenol coupling precursor to the (*R*)-semi-vioxanthin, combined with an auxiliary protein (Furtges et al., 2019). Overall, oxidases, particularly multicopper oxidase, perform a crucial function in PQ biosynthesis by dimerizing two phenol precursors.

The subsequent functional group modification of these dimer intermediates results in the wide diversity of PQs, for example, a flavin-dependent monooxygenase (ElcH) can transform hypocrellin into elsinochrome (Hu et al., 2019). In the absence of this flavin-dependent monooxygenase, an engineered *A. nidulans* generated hypocrellin through an intramolecular aldol reaction. A FAD-dependent monooxygenase, CTB7, has been proposed to catalyze the formation of the dioxepine ring of cercosporin (Chung and Liao, 2008); when the CTB7 gene was transferred into *Cercospora zeina*, it enabled cercosporin production (Swart et al., 2017), confirming the involvement of CTB7 in dioxepine ring formation. However, recent reports suggest that CTB9 and CTB10 also contribute to methylenedioxy bridge formation (de Jonge et al., 2018), which was confirmed by the crystal structures of CTB9 with various substrates and the subsequent site-directed mutagenesis study (Liu et al., 2022).

## Practical approaches to activate or stimulate the perylenequinone pathways of natural PQ-producing fungi

Gene-cluster-conservation analysis has revealed that many filamentous fungi contain one or more PQ BGCs (de Jonge et al., 2018). However, many of these BCGs are silent or are barely expressed, so these fungi may be potential sources of new or existing PQs. To exploit these potential PQs resources, novel physical, chemical and genetic approaches have been established.

### Strategies for modulating regulatory mechanisms of PQs biosynthesis

The regulation system of PQs biosynthesis is extremely complex and employs different modulation levels, including pathway-associated regulators, epigenetic regulators, and global regulators (Macheleidt et al., 2016). This section describes the corresponding regulatory mechanisms and strategies for PQs biosynthesis on basis of these regulatory systems.

#### Modulating transcription factors

**Figure 3:**
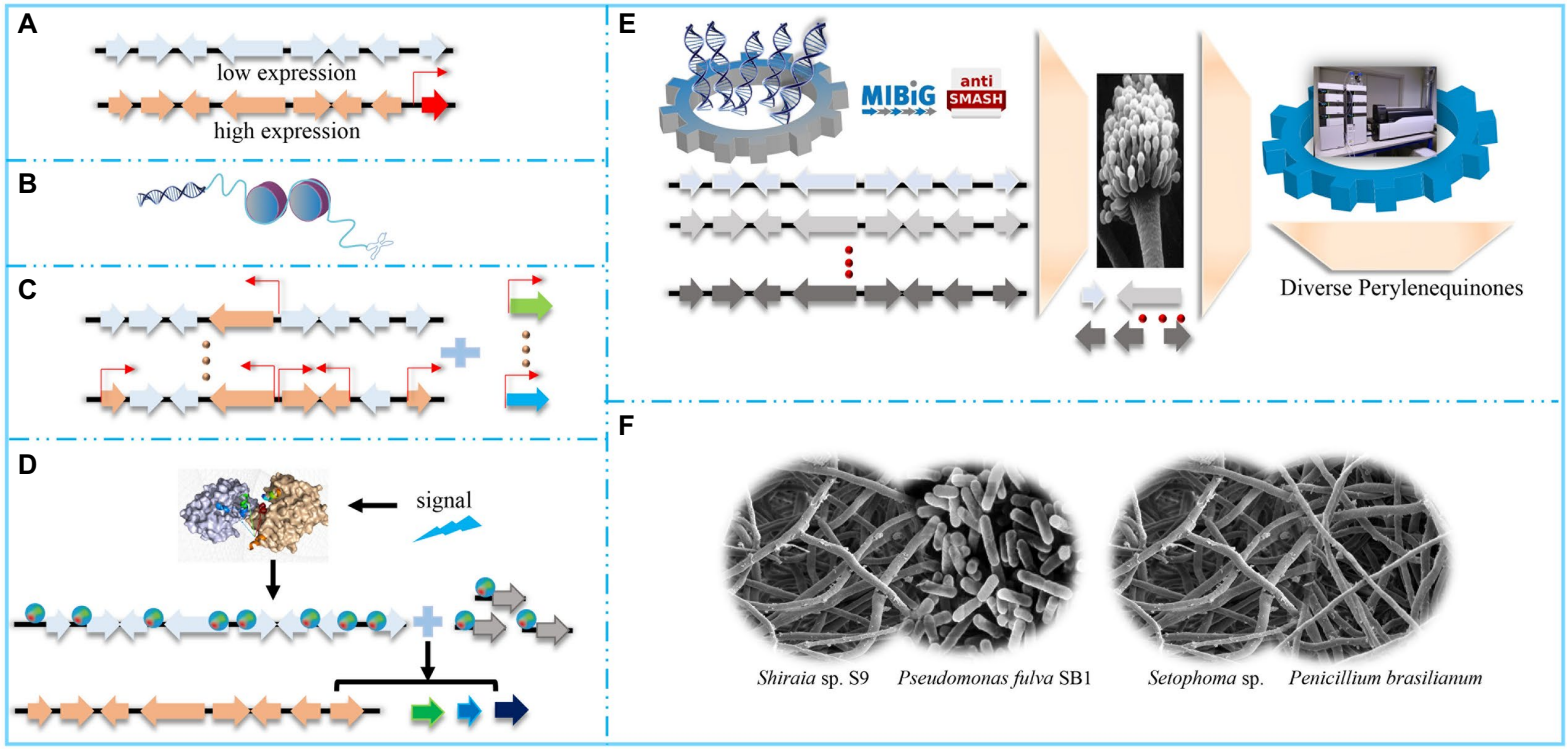
Diverse strategies to activate or broaden perylenequinone pools. **(A)** Activate the perylenequinone pathway by overexpressing pathway-specificity transcriptional factors. **(B)** Activate the perylenequinone pathway by engineering epigenetic regulators. **(C)** Enhance the perylenequinone pathway by increasing the central pathway. **(D)** Enhance the perylenequinone pathway by signal pathways. Environmental, chemical and physical signals trigger relevant global regulators, which regulate perylenequinone-linked genes by activating these genes. **(E)** Construct a diverse perylenequinone platform in tractable *Aspergillus* species. **(F)** Improve the perylenequinone pathway by microorganism co-culture, including bacteria-fungi co-cultures and fungi-fungi co-cultures.

PQ-associated transcription factors (TFs) are often distributed in the PQ biosynthesis gene clusters (BGCs) and are of the Zn_2_Cys_6_ zinc finger protein type (Figure 3A). They appear to coordinate gene expression in the PQ BGCs and regulate PQ production, so engineering these TFs has great potential for increasing PQ production efficiency. For example, bioinformatics analysis has indicated that the genome of wild-type *Parastagonospora nodorum* contains the elsinochrome BGC, but no elsinochrome production has been detected from this strain. Overexpressing the transcriptional regulator, elcR, has enabled transformants to generate elsinochrome by up-regulating elsinochrome-associated gene transcription (Chooi et al., 2017). Overexpressing elcR also enables elsinochrome production in transformants of *A. nidulans* (Hu et al., 2019), and enhancing gene expression of the hypocrellin-associated TF gene significantly increased hypocrellin production in the engineered *S. bambusicola* (Deng et al., 2020b).

#### Coordinating epigenetic regulators

Histone proteins act as scaffolds for nucleosome generation. Modifying these epigenetic regulators by genetic manipulation or chemical inhibitors (Figure 3B) can remodel chromatin cascades. Therein, these modifications trigger DNA methylation or acetylation, which stimulates PQ production by regulating gene expression in the PQ BGCs (Pfannenstiel and Keller, 2019). For example, treatment with suberoylanilide hydroxamic acid (Vorinostat, a histone deacetylase inhibitor) stimulates *C. cladosporioides* to produce several PQs, including cladochrome F and calphostin B (Williams et al., 2008). Trichostatin A (histone deacetylase inhibitor) stimulates *Colletotrichum fioriniae* to produce a PQ similar to cercosporin (de Jonge et al., 2018). 5-Azacytidine (DNA methylation inhibitor) down-regulates the light-response transcriptional factors (LaeA and VeA) involved in regulating hypocrellin biosynthesis in *S. bambusicola*, thus inhibiting gene transcription of the hypocrellin BGCs and markedly decreasing hypocrellin production (Ma et al., 2018).

#### Modulating environmental-response global signaling

Diverse environmental stimuli can also influence PQ production through global regulators (Figure 3), including AreA, CreA, PacC, and velvet complex, which can respond to changes in nitrogen sources, carbon sources, pH, and light, respectively. Unlike PQ-pathway TFs, these global regulators are not located in the PQ BGCs (You et al., 2008).

Among these environmental signals, light is the most influential factor for PQ biosynthesis. Therefore, light or dark signals have been widely used to modulate PQ production. Therein, the velvet complex (VeA and VelB) and laeA regulators respond to these light signals. In dthe arkness, VeA translocates to the nucleus and forms a heterodimer of VeA and VelB; and the constitutive orientation of LaeA promotes this velvet complex formation (Bayram et al., 2008). Therefore, different light conditions can influence velvet-related activities, such as secondary metabolic pathways. For example, switching between light and dark conditions enhances transcriptional levels of ROS-associated genes, thereby stimulating *S. bambusicola* to produce hypocrellin A (Sun et al., 2018), whereas *Cercospora nicotianae* forms cercosporin only under light conditions (You et al., 2008). Red light up-regulates the expression of genes for hypocrellin A and transmembrane transport, increasing hypocrellin production in the mycelia of *S. bambusicola* (Ma et al., 2019). Light stimulates mycelial growth of *Shiraia* sp. SUPER-H168, but suppresses hypocrellin biosynthesis. In contrast, darkness increases hypocrellin biosynthesis (Gao et al., 2018a).

AreA is a central regulator of nitrogen metabolism and consequently regulates PQ production. For instance, different nitrogen sources resulted in different levels of cercosporin production (You et al., 2008). Bioinformatics analysis has indicated that the promoter regions of cercosporin biosynthesis genes comprise one or more hypothetical GATA motifs, bounded by AreA (Chen et al., 2007), thus indicating that the genes in cercosporin BGCs are coordinated by AreA. Similarly, CreA stimulates PQ production by regulating carbon metabolism. Although PQ biosynthetic gene promoters contain no CreA binding sites (Chen et al., 2007), different carbon sources result in different levels of cercosporin production. Moreover, several PQ biosynthetic genes contain the PacC binding motif, indicating that cercosporin production could be influenced by pH (You et al., 2008).

### Regulation of PQ-associated genes from central metabolic pathways

The initial PQ precursor, acetyl-CoA is obtained from pyruvate through three enzymic steps (Figure 2). Acetyl-CoA carboxylase (ACC) transforms acetyl-CoA to malonyl-CoA, the extension unit for PQ biosynthesis; then PQ is biosynthesized by the central polyketide pathway (Figure 2). Therefore, modulating precursor supply gene expression and the main polyketide pathway increases PQ production (Figures 2, 3C). For example, ACC overexpression increases the acetyl-CoA substrate pool and stimulates polyketide production in various strains, such as *Streptomyces albus* (Lu et al., 2016), *Saccharomyces cerevisiae* (Wattanachaisaereekul et al., 2008), and *Aspergillus species* (Hasan et al., 2018). This suggests that increasing the polyketide precursor pool may be a feasible and generally applicable way to up-regulate production of targeted metabolites. Similarly, overexpressing two amylases can up-regulate the expression levels of carbon catabolic genes (such as *acc* and *pdc*) and hypocrellin pathway genes, resulting in three-fold higher hypocrellin yields in the engineered strains (Gao et al., 2018b). Regulating the genes from the central PQ pathway is another strategy to increase metabolite production. For instance, overexpressing a di-domain protein and a hydroxylase increases hypocrellin production three-and two-fold, respectively (Li et al., 2019). Overexpressing multicopper oxidase promotes the corresponding dimerization reaction and enhances the expression of hypocrellin-associated genes; hypocrellin production in the engineered transformants increases 5-fold compared with wild-type *S. bambusicola* (Deng et al., 2016b). The CRISPR-mediated transcriptional activation system can stimulate multigene activation in *A. nidulans* and should facilitate PQ production by improving related multi-gene expression (Roux et al., 2020).

### Strategies to coordinate endogenous signal-response pathways

Microorganism rapidly responds to the dynamic environmental signals for the continuous PQs formations through real-time signal pathways (Keller, 2019). Therefore, comprehending the transient signal translations and the relevant PQs regulatory mechanisms can facilitate PQs biosynthesis. Namely, PQs production might be improved by rationally engineering these signal pathways.

#### Modulation of the mitogen-activated protein kinase (MAPK) pathway

The MAPK signaling pathway is highly conserved in filamentous fungi and responds to signals from environmental stimuli (You et al., 2008). These signals are delivered by small GTPases to MAPK *via* phosphorylation reaction, thereby inducing translocation of MAPK to the nucleus, where it triggers specific TFs (Macheleidt et al., 2016). Notably, these processes coordinate with each other, suggesting that a crosstalk interaction exists, and may regulate PQ production. For example, the expression level of a MAPK-homologous protein in *Cercospora zeae-maydis* increases during cercosporin production, and a deletion mutant has decreased expression levels of cercosporin-related genes, thus inhibiting cercosporin biosynthesis. However, the MAPK complementation strain shows restoration of cercosporin production (Shim and Dunkle, 2003).

#### Regulating the calcium/calmodulin signal pathway

Calcium is a common cellular signal that regulates diverse physiological activities because intracellular calcium homeostasis is critical for PQ biosynthesis. Calcium homeostasis is modulated *via* several effectors (such as the calcium-binding protein, calmodulin) *via* one or more G proteins. Activating the G protein-dependent receptor of protein kinase A promotes the phosphorylation of RCS (regulator of calmodulin signaling) and its attachment to calmodulin, thereby modulating the intracellular Ca^2+^ concentrations and metabolite production. These processes involve crosstalk between the calcium/calmodulin signaling pathway and the MAPK pathway. Therefore, regulating these genes *via* genetic modification, Ca^2+^ addition, or Ca^2+^ complexation can influence PQ production. For example, pharmacological inhibitors block the Ca^2+^ channels, cause calcium ion disorder and inhibit cercosporin production in *Cercospora nicotianae* (Chung et al., 2003). Chemical Ca^2+^ inhibitors that down-regulate PQ biosynthetic gene expression also inhibit hypocrellin biosynthesis in *Shiraia* sp. Slf14 (Liu et al., 2018), whereas Ca^2+^ addition up-regulates PQ biosynthetic genes. For example, Ca^2+^ addition up-regulates the expression of cercosporin biosynthetic genes, including CRG1, CTB8, and CTB1, increasing cercosporin production (You et al., 2008). Similarly, Ca^2+^ addition increases hypocrellin production by up-regulating transcription of genes encoding Ca^2+^ ion sensors and increasing hypocrellin biosynthesis (Liu et al., 2018). Ca^2+^ addition could also ameliorate the repressive influence of inhibitors on gene expression and facilitate hypocrellin production (Liu et al., 2018).

#### Modulation of the endogenous ROS/oxidative stress-response systems

Oxygen is catalyzed to singlet oxygen and other ROS by the photoactivated PQs. Excessive ROS causes the disorder in cellular redox homeostasis and triggers oxidative stresses, which induce metabolic stagnation and cellular apoptosis (Montibus et al., 2015). However, *S. bambusicola* retains normal morphology and metabolic activity, even under high levels of oxidative stress and elevated hypocrellin production, indicating that these filamentous fungi have a very strong oxidative stress-response system, which protects these strains against the above ROS stresses (Deng et al., 2016a). Therefore, scientists have constructed the chassis for the continuous PQs biosynthesis by rationally engineering oxidative stress-response and regulation systems (Montibus et al., 2015). These processes mainly comprise two sections: (1) convert the toxic ROS to nontoxic compounds *via* global regulators; (2) deliver PQs out of the cells and reduce the ROS stress source by transporters.

AP1 is a well-known global regulator in the antioxidant system. High oxidative stress oxidizes the cysteine domains of the AP1, thereby forming disulfide bonds and activating AP1. The activated AP1 binds to nucleotide binding sites (5’-TTAGTCA-3′) of other stress-response genes and strengthens these gene expressions. Subsequently, superoxide radicals are converted into less harmful hydrogen peroxide, then to water, which ensures continuous PQs biosynthesis (Keller, 2015). For instance, AP1 overexpression enables cellular redox homeostasis and increases 6-fold hypocrellin productions by up-regulating expressions of genes, such as superoxide dismutase and catalase (Deng et al., 2020b). Similar to AP1, crg1 also improves the expression of genes, which are involved in the cercosporin biosynthesis and the resistance to cercosporin toxicity (Chung et al., 2003).

Transporter engineering is another promising approach to export the biosynthesized PQs from producer cells, reduce oxidative stress, and facilitate the high yielding PQ production (Chen et al., 2007; Newman and Townsend, 2016). Therein, ATP hydrolysis enables ATP-binding cassette (ABC) transporters to deliver small molecules and macromolecules by concentration gradients, whereas major facilitator superfamily (MSF) transporters can transfer small molecules by chemiosmotic ion differences (Keller, 2015). Therefore, ABC and MSF transporters can remove toxic molecules from filamentous fungi, thus enabling high PQs production and protecting the producer organisms against PQ toxicity. For instance, overexpression of ABC and MSF transporters increases about 5-and 4-fold hypocrellin production, respectively (Deng et al., 2020b). Photoactivated-cercosporin metabolites increase the expression of ABC and MSF transporters, and overexpression of these transporters enables the normally PQ-sensitive *Neurospora crassa* to maintain normal morphology during photoactivated-cercosporin treatment (Beseli et al., 2015). Upregulation of a uracil transporter and a zinc transporter has been also determined to stimulate the PQs biosynthesis (Deng et al., 2017c).

### Construction of a diverse PQ platform in the tractable *Aspergillus* species

Genes from the PQ pathway are highly homologous, and other non-conserved genes may be useful for modifying PQ functional groups and generating new combinations thereof (de Jonge et al., 2018; Ebert et al., 2019). Modifying genes for functional group modification should be an effective strategy to construct a diverse PQ platform. For example, *Cryphonectria parasitica* can generate various complex pigments through the polyketide pathway, and transformants with *Cppks1* also produced a non-natural phleichrome (So et al., 2015). Recently, our group has constructed an efficient CRISPR system (Deng et al., 2017a), which can be used to integrate or replace functional group modification genes to generate novel PQs.

Reconstructing PQ BGCs in tractable filamentous fungi is another feasible approach to generate novel PQs (Figure 3E) because these strains have been engineered as auxotroph. Thus, transforming plasmids (containing the corresponding prototrophic genes) into these mutants can facilitate the growth of positive strains on selective media (Chiang et al., 2013). In addition, PQ biosynthesis genes are colinear in the PQ BGCs (Blin et al., 2017), containing nucleotides larger than 30 kb (Hu et al., 2019). These longer sequences require efficient methods to express partial or whole pathways; therefore, a fungal artificial chromosome (FAC) approach has been designed to randomly transfer the relevant genomic regions (up to 300 kb), thereby facilitating the transfer of the intact PQ BGCs into the expression plasmids (Tsunematsu et al., 2013; Bok et al., 2015). These plasmids contain bacterial and filamentous fungal replication origins, which ensure successful pathway construction in *E. coli* and target metabolite expression in *Aspergillus* species. After expression in *Aspergillus* species, high throughput detection tools are essential to confirm the presence of the target products. Liquid chromatography/mass spectrometry (LC/MS) was previously the method of choice to analyze *Aspergillus* metabolomics. Molecular networking and in-silico MS/MS fragmentation tools have further emerged as efficient methods to exploit complex metabolite profiles and the relevant PQ derivatives in filamentous fungi (Allard et al., 2016). The recently developed Global Natural Products Social Molecular Networking (GNPS; Wang et al., 2016) is an open-access data analysis tool, offering diverse raw, or assigned MS/MS libraries, which facilitates continuous dereplication and reliable product confirmation. In addition, a metabolomics-scoring strategy has been established to determine the FAC-encoded metabolites of different *Aspergillus* species, based on GNPS (Clevenger et al., 2017). For instance, the elsinochrome pathway in *Aspergillus nidulans* has been deconstructed and the target metabolites have been detected; engineering *Aspergillus nidulans* with a flavin-linked monooxygenase generates elsinochrome, whereas the transformants without this monooxygenase produce hypocrellin (Hu et al., 2019). Given the substantial homology between the elsinochrome and cercosporin BGCs, ElcE and ElcG have been used as reference genes, and two oxidases, i.e., FAD-dependent CTB5 from *Cercospora nicotianae* and Cz_CTB12 from *Cercospora zeae-maydis,* have been identified. Through the gene swap principle, ElcE and ElcG of *A. nidulans* have been replaced with CTB5 and Cz-CTB12, and the modified transformants generates the PQ core ring structure (Hu et al., 2019). These findings indicate the possibility of generating diverse PQ structures by reconstructing the pathways in *Aspergillus* species.

A droplet microfluidic platform has been developed in *Aspergillus* species *via* complex digital microfluidic/channel-based droplet chips (Gach et al., 2016). Similarly, liquid-handling robots can largely eliminate repetitive sample handling and facilitate very large-scale automated biochemical experiments; for example, liquid-handling robots have achieved 100% genomic editing frequency in *Aspergillus* (Kuivanen et al., 2019). These two systems offer the possibility to reconstruct the PQ pathway in *Aspergillus* species in a fully automated manner.

### Other approaches to stimulate endogenous perylenequinone biosynthesis

Except for endogenously genetic regulation, other methods, such as physical and chemical strategies can also stimulate PQs biosynthesis by increasing expressions of genes that are involved in hypocrellin biosynthesis, secretion, and auto-resistance (Sage and Shikazono, 2017; Sun et al., 2017).

#### Irradiation

Irradiation causes extensive ionization of DNA, which results in multiple-site damage, such as DNA lesions, abasic sites, or double-strand breaks (Sage and Shikazono, 2017), which can trigger variations in fungal morphology and secondary metabolites. Subsequent high-throughput screening can isolate candidate mutants with higher PQ production, or enable the production of PQs with structural changes. Therefore, irradiation is a common method to promote or modify PQ biosynthetic pathways. For example, gamma irradiation of wild-type *S. bambusicola* spore causes 80% lethality and a 35% positive mutant frequency, which increases hypocrellin production 4-fold (Liu et al., 2016). Low-intensity ultrasound also increases cell membrane permeability, antioxidant enzyme activity, and gene expression of the hypocrellin BGC (Sun et al., 2017). Therein, the up-regulation of hypocrellin biosynthesis, secretion, and auto-resistance increased the hypocrellin yield by 1.8-fold (Sun et al., 2017).

#### Chemical elicitors

Chemical elicitors are environmental signals which can trigger PQ gene expression, thereby stimulating PQ biosynthesis. For example, the surfactant, Triton-X 100, triggers hypocrellin production, whereas SDS and Tween 40 do not (Cai et al., 2011). Transcriptomic analysis has indicated that Triton-X 100 up-regulates the expression of genes encoding membrane permeability, hypocrellin secretion, and hypocrellin biosynthesis (Lei et al., 2017); i.e., Triton-X 100 triggers hypocrellin production in mycelia and enhances transfer to the medium. The rare-earth element, lanthanum also stimulated hypocrellin production in *S. bambusicola* by enhancing PQ-associated gene expression (Lu et al., 2019). Microbial elicitors can improve membrane permeability and enhance PQ-associated gene expression; therefore, they provide another means of facilitating PQ biosynthesis and transportation. For example, two diketopiperazines from *Epichloe typhina* have been found to facilitate phleichrome production in *C. phlei* (So et al., 2015). An elicitor from *Aspergillus niger* also enhances hypocrellin production by increasing the production of signaling molecules, including salicylic acid and nitric oxide (Du et al., 2015; Ma et al., 2021).

#### Co-culture with other organisms

Microbial communities are widespread in nature and relationships among the different species in the community comprise symbiosis, antagonism, and competition, which are mainly mediated by secondary metabolites. Therefore, microorganism co-culture is evolving as an efficient approach to increase the diversity of secondary metabolites in general, particularly for PQs (Figure 3F; Bertrand et al., 2014). Microorganism co-culture involves bacteria-bacteria, bacteria-fungi, and fungi-fungi co-culture. Co-culture facilitates the expression of enzymes that produces metabolite precursors and may also stimulate epigenetic modification of the target host; therefore, co-culture is an effective strategy to stimulate PQ biosynthesis. For example, co-culture of the ΔCTB2 and ΔCTB3 mutants does not generate any cercosporin, whereas red cercosporin is produced from co-culture of the ΔCTB1 and ΔCTB2 mutants (Chen et al., 2007); co-culture compensates for pathway deficiencies in the single mutants and produces an intact PQ pathway. Similarly, neither of the ΔCTB1 and ΔCTB3c mutants was able to produce cercosporin; however, a co-culture of these mutants does produce cercosporin (Chung and Liao, 2008). Similarly, stemphyperylenol and derivatives can be produced by co-culturing *Setophoma* sp. with *Penicillium brasilianum* (Bazioli et al., 2020). Co-culture with *Pseudomonas fulva* SB1 facilitates hypocrellin and elsinochrome production in *Shiraia* sp. S9 (Ma et al., 2019); co-culture stimulates the expression of genes, thereby contributing to PQ biosynthesis and secretion. In addition, *Pseudomonas fulva* SB1 facilitates transient ATP release from *Shiraia* sp. S9 (Li et al., 2021). The extracellular ATP can function as a damage-associated-molecular pattern (DAMP) and this DAMP signal is associated with interactions between bacteria and fungi. Moreover, extracellular ATP is associated with Ca^2+^ signaling and ROS generation, which trigger fungal conidiation and PQ biosynthesis (Li et al., 2021). Endophytic bacteria, including *Bacillus velezensis* B04 and *Lysinibacillus* sp. B15, stimulate cercosporin biosynthesis (Zhou et al., 2021).

#### Drug resistance screening

The photo-activated ROS by PQs can trigger diverse cellular apoptosis. These candidate PQs can also circumvent the multidrug-resistance resistance of pathogenic organisms. Therefore, the chemicals with antimicrobial activities could be used as an efficient strategy to screen novel PQs from fungal extractions. For example, an extract of *Phialocephala fortinii* growth medium could reverse azole resistance in *C. albicans* (Xie et al., 2016). The active component of the extract is phialocephalarin B, which up-regulates the expression of drug-delivery genes, thereby resulting in the reversal of azole resistance (Xie et al., 2016).

## Conclusions and perspectives

As excellent photosensitizers, various PQs have been applied in the medical, food, agricultural, and manufacturing fields. These extensive application prospects have stimulated the development of new and improved technologies for the discovery of new PQs and the elucidation of PQ biosynthetic pathways. Evolving sequencing tools have identified many PQ biosynthetic gene clusters (BGCs) in fungal genomes. Bioinformatics software such as AntiSMASH and the MIBiG repository facilitates the discovery of diverse PQs, based on their highly conserved BCGs. Gene editing technologies, particularly the automated CRISPR system, facilitate the automated high-throughput capability to elucidate and confirm these BGCs. Enzymatic conversion experiments are an essential confirmatory test for PQ genes or pathways. The data obtained from the above techniques have enabled higher PQ yields, titers, and productivity. Modifying only one gene decreases the need for complex genetic mutagenesis for the reassembly of entire PQ pathways and the improvement of PQ production. Regulation of signaling pathways is essential to balance the complex cellular metabolic flux and biosynthetic precursor supply and optimize PQ production. These coordination approaches are complex and difficult to implement in many fungal PQ strains, particularly wild-type organisms. However, reconstructing the PQ biosynthetic pathway in tractable *Aspergillus* species is a feasible and efficient strategy to obtain a high PQ yield. These evolving technologies in tractable *Aspergillus* species should enable the automation of PQ de-and re-construction. These methods involve four stages: (1) the computer-aided design stage can elucidate or associate new PQ pathways, (2) the construction stage can assemble target PQ pathways in tractable *Aspergillus* species, (3) the evaluation stage can determine metabolic bottlenecks and blockages, and (4) the optimization stage can fine-tune the PQ biosynthetic pathways and precursor supply from central metabolism, and facilitates the production of the desired products.

## Author contributions

HD and YC designed this review. HD wrote this review. XL and JL draw the figures and tables. XZ and T-PF helped to revise the manuscript. All authors reviewed, contributed to the manuscript, and approved the submitted version.

## Funding

This work was supported financially by the Natural Sciences Foundation of China (32201203), the Natural Sciences Foundation of Guangdong Province (2021A1515110263), and the Natural Sciences Foundation of Jiangsu Province (BK20210471).

## Conflict of interest

The authors declare that the research was conducted in the absence of any commercial or financial relationships that could be construed as a potential conflict of interest.

## Publisher’s note

All claims expressed in this article are solely those of the authors and do not necessarily represent those of their affiliated organizations, or those of the publisher, the editors and the reviewers. Any product that may be evaluated in this article, or claim that may be made by its manufacturer, is not guaranteed or endorsed by the publisher.
