# Supplementary material for: Advances and perspectives on perylenequinone biosynthesis
Source: Front Microbiol. 2022 Dec 20;13:1070110. doi: 10.3389/fmicb.2022.1070110 (PMC9808054; doi:10.3389/fmicb.2022.1070110)
Supplement: Supplementary file 1 [file Data_Sheet_1.docx]

**Supplementary Information**

# Advances and perspectives on perylenequinone biosynthesis

Huaxiang Deng^1,2*^, Xinxin Liang^2^, Jinbing Liu^3^, Xiaohui Zheng^4^, Tai-Ping Fan^5^, Yujie Cai^2*^

^1^ Center for Synthetic Biochemistry, Shenzhen Institute of Synthetic Biology, Shenzhen Institute of Advanced Technology, Chinese Academy of Sciences, Shenzhen 518055, China

^2^ The Key Laboratory of Industrial Biotechnology, Ministry of Education, School of

Biotechnology, Jiangnan University, 1800 Lihu Road, Wuxi, Jiangsu 214122, China

^3^ School of Marine and Bioengineering, Yancheng Institute of Technology, Yancheng, Jiangsu, 224051, China

^4^ College of Life Sciences, Northwest University, Xi’an, Shanxi 710069, China

^5^ Department of Pharmacology, University of Cambridge, Tennis Court Road Cambridge CB2 1PD, United Kingdom

^*^Corresponding author: yjcai@jiangnan.edu.cn (Yujie Cai); denghxiang@163.com (Huaxiang Deng)

**Contents**

**Figure S1.** The basic core structure of perylenequinones.

**Figure S2.** Structures of Phaeosphaerins A-F.

**Figure S3.** Structures of Calphostin, Cladochromes, Phleichrome, Cercosporin, Elsinochrome, and Hypocrellin.

**
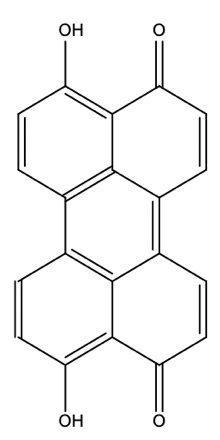
Figure S1.** The basic core structure of perylenequinones.

**
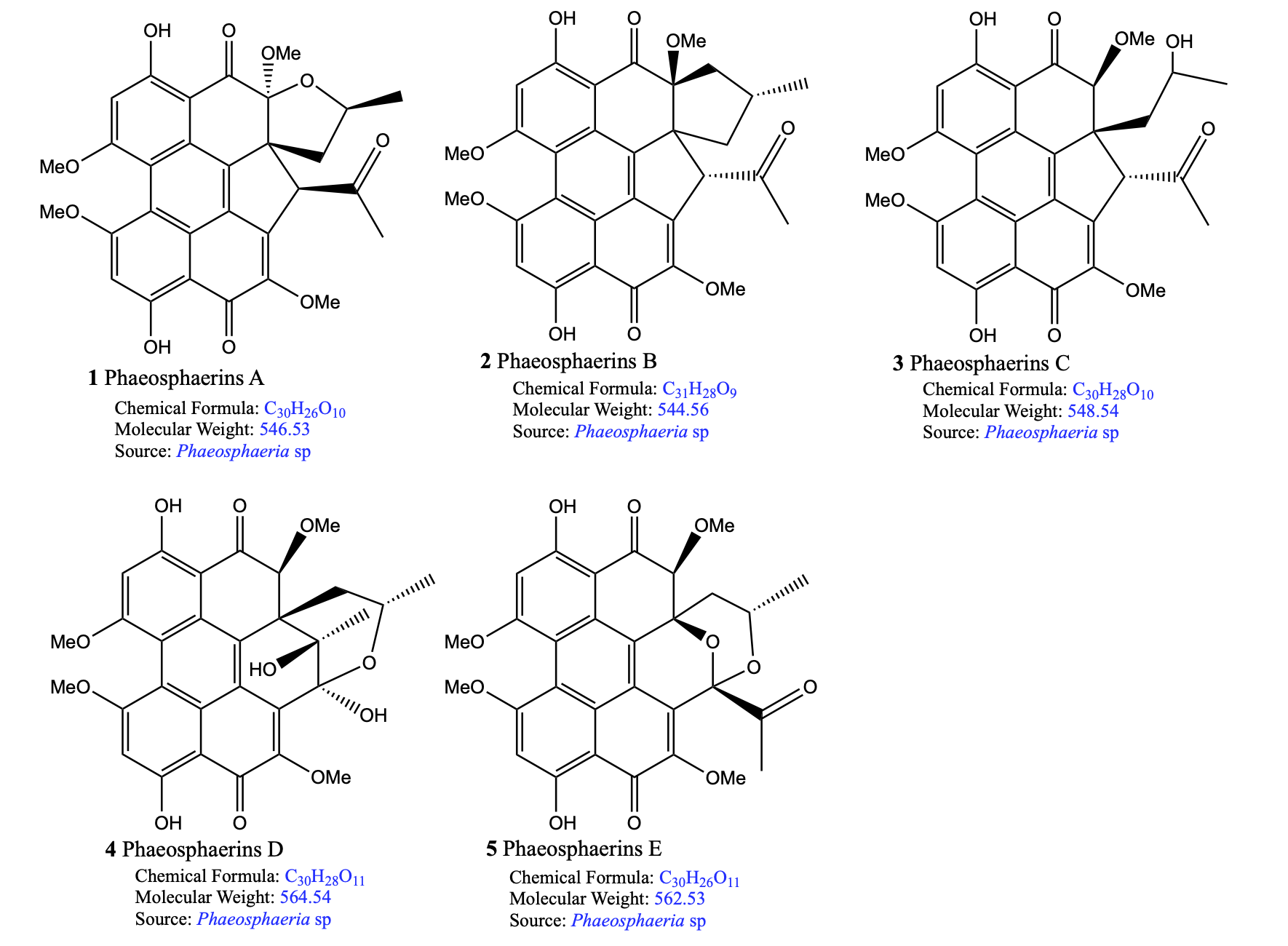
**

**Figure S2.** Structures of Phaeosphaerins A-F.

**
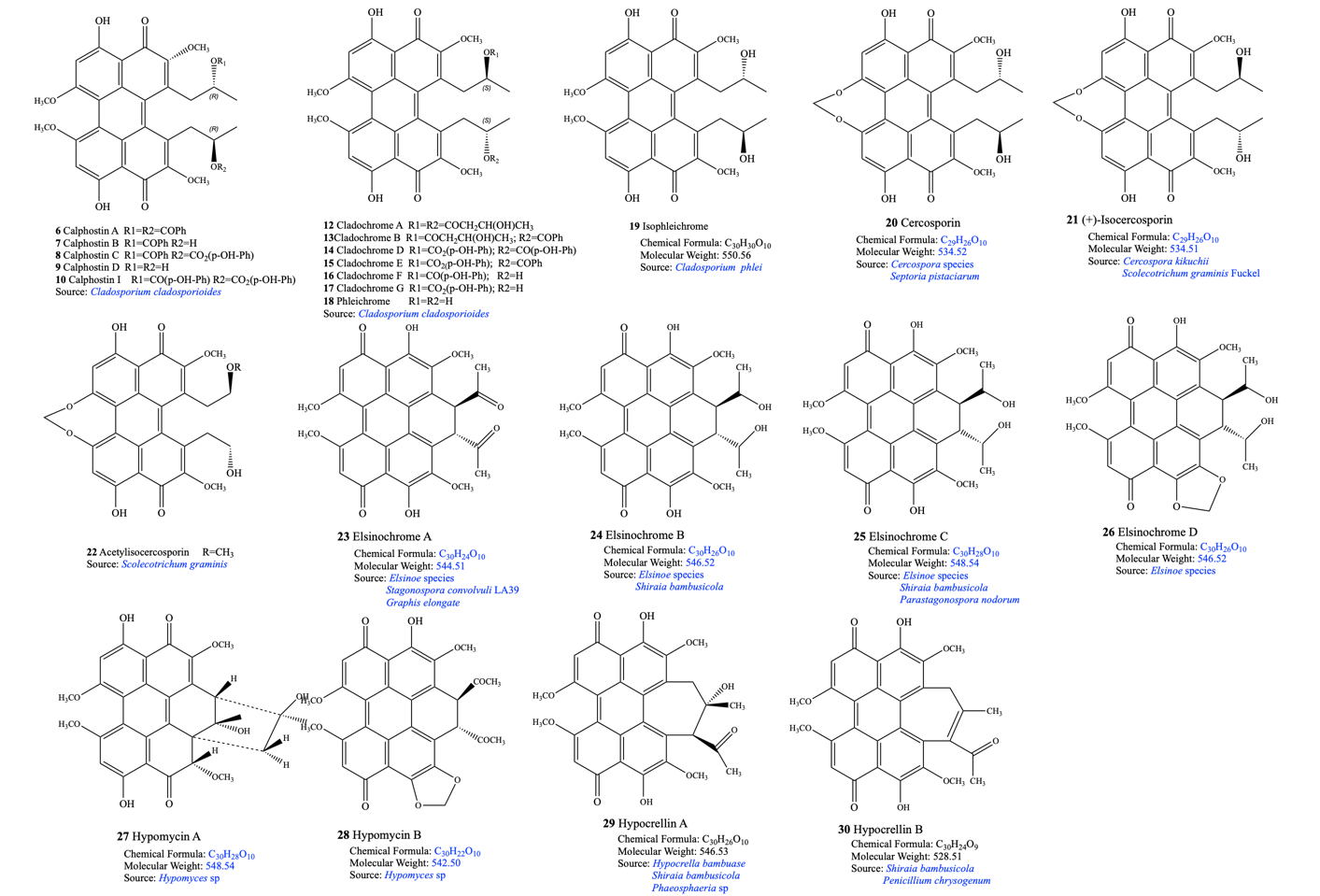
Figure S3.** Structures of Calphostin, Cladochromes, Phleichrome, Cercosporin, Elsinochrome, and Hypocrellin.
